# Aggravated Gut Microbiota and Metabolomic Imbalances Are Associated with Hypertension Patients Comorbid with Atrial Fibrillation

**DOI:** 10.3390/biom12101445

**Published:** 2022-10-09

**Authors:** Chen Fang, Kun Zuo, Yuan Fu, Xiaoming Zhu, Jing Li, Jiuchang Zhong, Li Xu, Xinchun Yang

**Affiliations:** Heart Center & Beijing Key Laboratory of Hypertension, Beijing Chaoyang Hospital, Capital Medical University, Beijing 100020, China

**Keywords:** atrial fibrillation, hypertension, metagenome, metabolome, saturated fatty acids, unsaturated fatty acids

## Abstract

Disordered gut microbiota (GM) as the co-contributor of atrial fibrillation (AF) and hypertension (HTN) might be associated with AF risk in HTN. This study aimed to explore the altered GM community and metabolic patterns between 27 HTN patients with AF (HTN-AF) and 27 non-AF HTN patients through fecal metagenomic and serum metabolomic analysis. Compared to non-AF HTN patients, significant microbial alterations (*p* = 0.004), including increased microbial diversity (*p* < 0.05), shifted enterotype dominated by *Prevotella* to *Bacteroides*, and abundant disease-linked genera *Ruminococcus*, *Streptococcus*, *Veillonella*, *Dorea*, and *Enterococcus*, were observed in HTN-AF patients. A species-based random forest prediction model was associated with the risk of AF occurrence in HTN patients. Furthermore, GM metabolic profiles dramatically differed between HTN and HTN-AF patients, especially the imbalance of saturated and unsaturated fatty acids. In HTN-AF patients, circulating palmitic acid and arachidonic acid levels were significantly elevated, while the levels of tetracosahexaenoic acid, oleic acid, linoleic acid, and stearic acid were decreased (*p* < 0.001, VIP > 1), mediating 85.99% of gut microbial indirect effects on AF (*p* < 0.001). Thus, our findings preliminarily indicated that exacerbated dysbiosis of GM and relevant metabolites was associated with high AF susceptibility and might be a potential target for AF prediction and prevention in HTN.

## 1. Introduction

Atrial fibrillation (AF), the most common cardiac arrhythmia, portends a 2- to 5-fold increase in the risk of stroke, heart failure (HF), and mortality [1]. As a major contributor involved in AF pathogenesis, hypertension (HTN) explains more than 20% of AF cases [2]. In the Framingham Heart Study, HTN increases AF risk by 50% in men and 40% in women [3]. Notably, the SPRINT cohort study reports that aggressive control of blood pressure was not associated with reduced risk of AF incidence in HTN [4]; thus, the residual risk factors need to be identified and addressed between HTN and AF.

Accumulating evidence demonstrates that trillions of gut microbiota (GM) colonizing the human gastrointestinal tract generate bioactive metabolites, impacting host homeostasis, and function as the largest endocrine organ in the body [5]. In particular, disturbed gut microbial structure and function, and its derived metabolites, play pivotal roles in the progression of cardiovascular disease, including HTN and AF [6,7]. Aberrant GM composition and related metabolites, including trimethylamine N-oxide (TMAO) and lipopolysaccharides (LPS), contribute to HTN and its complication (e.g., chronic kidney disease, stroke, heart failure, and myocardial infarction) [7,8]. Our previous studies revealed associations between GM dysbiosis, metabolomic alteration and AF type, duration, post-ablation recurrence, and thromboembolic risk [9,10,11,12]. Although the importance of gut microbial disorders has been reported in HTN and AF separately, the role of GM in AF susceptibility in HTN patients remains unclear. 

**Figure 1:**
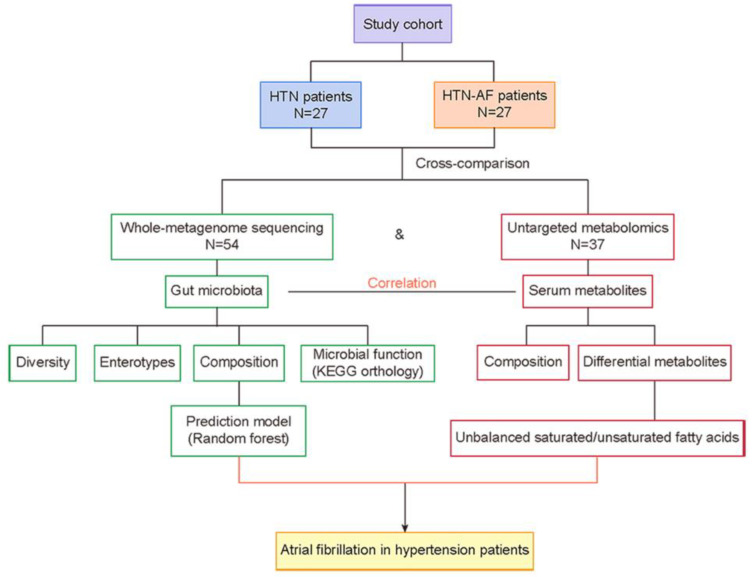
Overview of the workflow. AF, atrial fibrillation; HTN, hypertension.

Therefore, this study assessed the GM and metabolic features in HTN patients with or without AF, based on high-throughput metagenomic and metabolomic analyses, and provided an initial understanding of GM dysregulation in HTN patients coupled with AF occurrence. The workflow is presented in Figure 1.

## 2. Materials and Methods

### 2.1. Study Cohort

A total of 27 HTN patients with AF (HTN-AF) and 27 non-AF HTN patients were included from our previous study [13]. The exclusion criteria included heart failure, coronary heart disease, structural heart disease, inflammatory bowel diseases, irritable bowel syndrome, autoimmune diseases, liver diseases, renal diseases, cancer, or the use of antibiotics or probiotics in the last month. Clinical characteristics were collected by face-to-face surveys and medical records. The research protocol was approved by the ethics committee of Beijing Chaoyang Hospital Affiliated to Capital Medical University and Kailuan General Hospital. All the participants signed informed consent forms.

### 2.2. GM Analysis by Metagenomics and Metabolomics

In the current study, 54 whole-metagenome sequencing data and corresponding 37 metabolomic data were available from our previous published datasets [13]. Metagenomic sequencing, gene catalog construction, gene prediction, taxonomic and functional annotation, abundance profiling, and enterotype analysis were performed following the previously described procedures. For metabolomics measurements, liquid chromatography–mass spectrometry (LC/MS) was carried out. Methods for feature extraction, data normalization, and identification of compounds were performed as we previously described [13]. Significantly discriminate compounds between groups were identified by *p* < 0.05 and variable importance for the projection (VIP) > 1, based on the normalized peak intensities using *t*-test and orthogonal partial least squares discriminant analysis (OPLS-DA).

### 2.3. Statistical Analysis

Continuous variables with normal and nonnormal distributions were presented as mean ± standard deviation (SD) and median (first quartile, third quartile) and analyzed by Student’s *t*-test or Mann–Whitney test to assess differences between two groups, respectively. Categorical data were compared using the Chi-square test and presented as a percentage. Pielou evenness, Shannon, and Simpson indexes were calculated with R software (version 3.3.3, R Foundation for Statistical Computing, Vienna, Austria) to evaluate gut microbial alpha-diversity. Principal component analysis (PCA) was analyzed using the FactoMinR package, and principal coordinate analysis (PCoA) was performed by vegan and ape package in R software (version 3.3.3) to investigate differences in GM and serum metabolites between two groups. The partial least squares discriminant analysis (PLS-DA) was carried out using the SIMCA-P software to cluster the sample plots across groups. Differential abundance of genera, species, and KEGG orthology (KO) was tested by the Wilcoxon rank sum test, and *p* values were corrected for multiple testing with the Benjamin–Hochberg method. LEfSe online analysis tool was performed to assess the altered GM between groups. Spearman’s correlation analysis calculated the correlation between metabolic and microbiome abundances. A prediction model was constructed using microbial markers selected by the random forest classifier in R software (version 3.3.3), which was further applied for ROC analysis to test the predictive potential of AF. Partial least squares structural equation modeling (PLS-SEM) was used to evaluate the interaction of GM, metabolites, and AF occurrence in HTN patients by SmartPLS 3.0 software (SmartPLS GmbH, Hamburg, Germany). All statistical tests were 2-sided, and *p * <  0.05 was considered significant. 

## 3. Results

### 3.1. Baseline Characteristics of the Study Cohort

The present study included 27 HTN patients with AF and 27 non-AF HTN patients. There was no significant difference between HTN and HTN-AF patients in terms of body mass index (BMI), serum creatinine (sCr), triglyceride (TG), low-density lipoprotein (LDL), alanine aminotransferase (ALT), and fasting blood glucose (FBG), except for total cholesterol (TC), diabetes mellitus (DM), male gender and age. The TC levels of all participants were within normal levels. The specific baseline characteristics were shown in Table 1.

### 3.2. Altered Gut Microbial Diversity and Enterotype Distribution in HTN Patients with AF

**Figure 2:**
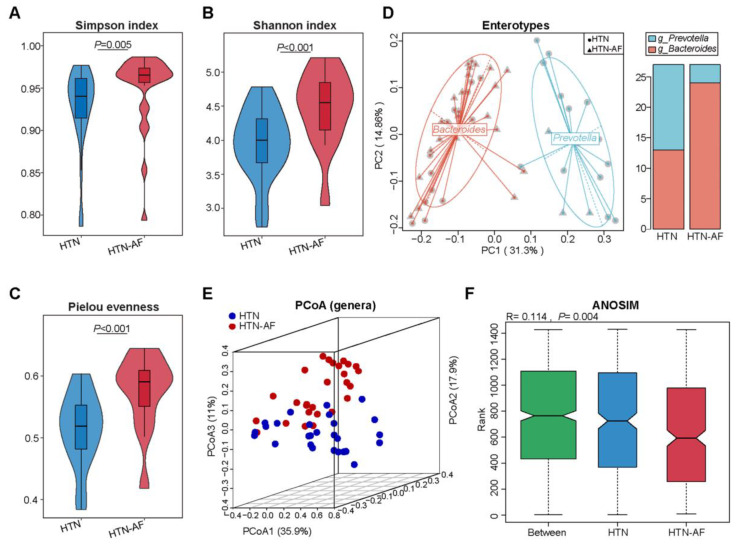
Elevated microbial diversity and altered GM structure in HTN patients with AF. (**A**–**C**) Differences in alpha-diversity of gut microbiota between HTN and HTN-AF groups, including Simpson index (**A**), Shannon evenness (**B**), and Pielou index (**C**). Wilcoxon rank sum test. (**D**) Left: 54 samples were clustered into two enterotypes dominated by *Bacteroides* (red) and *Prevotella* (blue), respectively, using principal coordinate analysis (PCoA) of Jensen–Shannon divergence values at the genus level. Right: the percentage of HTN and HTN-AF samples distributed in two enterotypes. (**E**) PCoA plots based on genus showed the variant gut microbial structures in the two groups. (**F**) ANOSIM analysis of GM between the two groups.

To determine alterations in gut microbial structure between HTN and HTN-AF patients, we analyzed the alpha- and beta-diversity at the genus level based on metagenomic sequencing data. Compared with the HTN group, the HTN-AF group had a significantly elevated alpha-diversity of gut microbes, including the Simpson index, Pielou evenness, and Shannon index (Figure 2A–C), indicating the disordered profile of bacterial composition in HTN patients comorbid with AF. Meanwhile, gut microbial enterotype features were identified using the partitioning around medoid clustering method, and PCoA based on the Jensen–Shannon distance was performed to cluster the 54 samples into two distinct enterotypes (Figure 2D). Enterotypes 1 and 2 were dominated by genus *Bacteroides* and *Prevotella*, respectively. The results revealed a higher proportion of enterotype 1 in HTN-AF patients, while there was more enterotype 2 in non-AF HTN patients (Figure 2D). Thus, HTN patients with AF tended to have a *Bacteroides*-dominated enterotype. 

### 3.3. Distinct Gut Microbial Compositions between HTN and HTN-AF Groups

**Figure 3:**
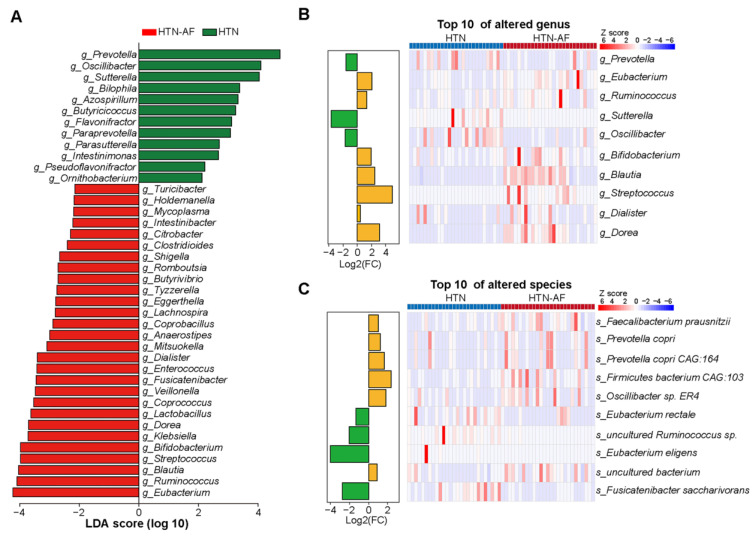
Differential GM compositions between HTN and HTN-AF patients. (**A**) Histogram of linear discriminant analysis (LDA) combined with effect size (LEfSe) analysis with absolute LDA scores (log10) > 2 and *p* values < 0.05. (**B**,**C**) Histogram and heat map illustrating the log2 fold-change (HTN-AF/HTN) and relative abundance of the top 10 significantly altered genus (**B**), and species (**C**) in HTN and HTN-AF groups, respectively.

Furthermore, significant differences in gut microbial composition were detected in HTN and HTN-AF patients via PCoA and ANOSIM analysis (R = 0.114, *p* = 0.004) (Figure 2E,F). A total of 1224 species and 249 genera were dramatically different between the two groups, according to the Wilcoxon rank sum test. The top 10 relative abundances of differential genera and species were presented in Figure 3B,C. Using LEfSe analysis, 40 high-dimensional biomarkers were discovered, of which 12 discriminant genera were abundant in the HTN group and 28 genera were enriched in the HTN-AF group (Figure 3A). Multivariate analysis by linear models (MaAsLin) was used to adjust for potential confounders (such as TC, gender, DM, and age), verifying that the remarkable differences in these genera between the two groups were mediated by AF rather than baseline clinical factors (Figure S1 and Table S1). Notably, the host-disrupting genera *Ruminococcus*, *Streptococcus*, *Veillonella*, *Dorea*, and *Enterococcus* were abundant in HTN patients with AF (Figure 3A) [13,14,15,16,17,18]. These results suggested that aggravated gut microbial dysbiosis was involved in the AF occurrence of HTN patients. 

### 3.4. Disturbance of GM Functions in HTN-AF Patients

**Figure 4:**
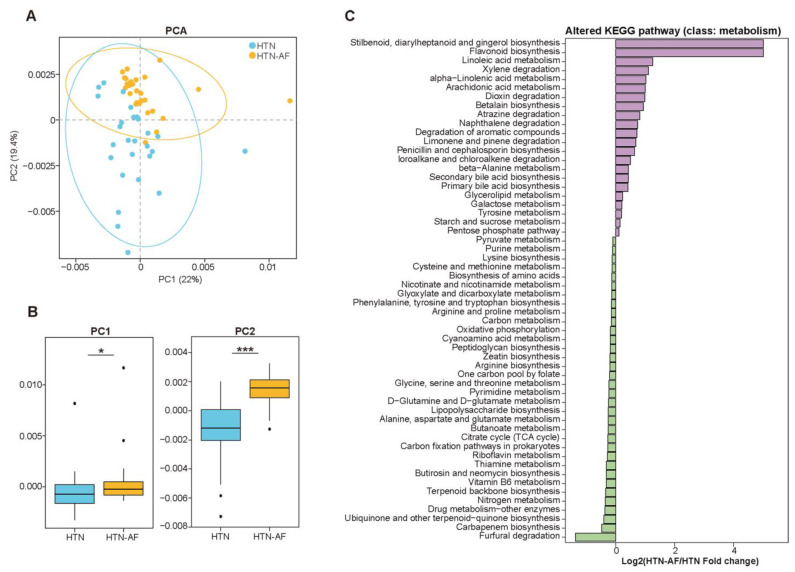
The shift in gut microbial function in HTN-AF patients. (**A**,**B**) PCA score plots based on annotated KEGG orthology (**A**) and related comparison of PC1 and PC2 (**B**) revealed the altered gut microbial function between the two groups. (**C**) Log2 fold-change of differential KEGG pathway (class: metabolism) between HTN and HTN-AF groups (*p* < 0.05). Wilcoxon rank sum test. *, *p <* 0.05; ***, *p* < 0.001.

According to the Kyoto Encyclopedia of Genes and Genomes (KEGG) database, we evaluated gut microbial functions in the two groups, annotating 5119 KOs and 312 KEGG pathways. PCA based on KOs distinguished HTN-AF patients effectively from non-AF HTN patients (Figure 4A,B), and ANOSIM analysis further confirmed the differed KOs in the two groups (R = 0.221, *p* = 0.001) (Figure S2). Meanwhile, Figure 4C presented 56 KEGG pathways (class: metabolism) with significant differences (*p* < 0.05), including stilbenoid, diarylheptanoid and gingerol biosynthesis, flavonoid biosynthesis, linoleic acid metabolism, alpha-linolenic acid metabolism, arachidonic acid metabolism, secondary bile acid biosynthesis, and primary bile acid biosynthesis, etc. Although the functional annotation analysis was predictive, these findings indicated that disordered GM promoted AF occurrence in HTN patients, possibly mediated by GM-dependent metabolic dysregulation. 

### 3.5. AF Occurrence in HTN Patients was Related to Changed Serum Metabolomics

**Figure 5:**
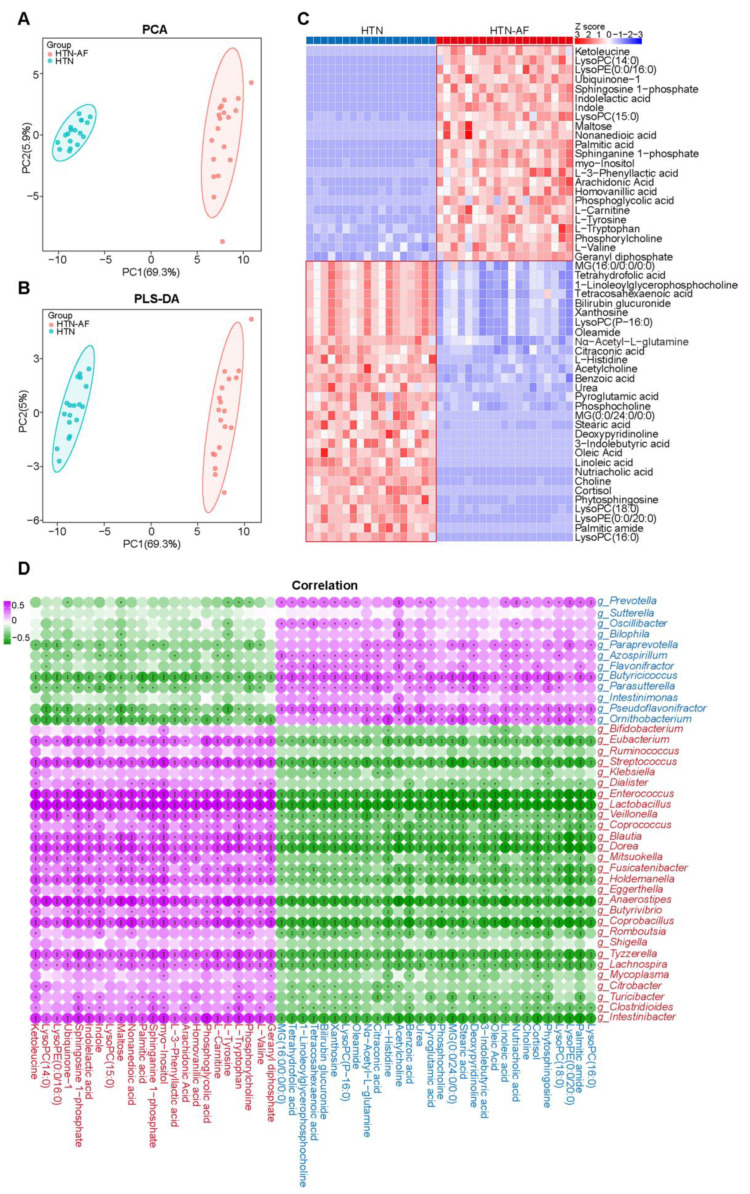
Altered metabolic signatures in HTN-AF patients. (**A**,**B**) PCA and PLS-DA score plots based on the serum metabolic profiles depicting the metabolic differences between HTN and HTN-AF groups. (**C**) Heat map describing the relative abundance of dramatically shifted serum metabolites in the two groups (*p* < 0.05, VIP > 1). Abundance profiles were converted to Z-scores via average abundance subtraction and division by the standard deviation. Negative (blue) and positive (red) Z-scores reflected lower and higher row abundance levels than the mean, respectively. (**D**) Spearman’s correlation analysis revealed the correlation between altered genera and metabolites in the two groups. Purple, a positive correlation; green, a negative correlation; red, genera or metabolites enriched in HTN-AF group; blue, genera or metabolites enriched in HTN group; *, *p <* 0.05; **, *p* < 0.01; ***, *p* < 0.001.

Considering that some of the metabolites in the circulation derived from intestinal microbial fermentation and distinct metabolic functions in the HTN-AF and HTN groups, we analyzed LC–MS-based untargeted serum metabolome profiles to define GM metabolic dysfunction further. Multivariate analysis of the PCA and PLS-DA revealed the dramatically different distribution of serum metabolites between the two groups (Figure 5A,B). As shown in Figure 5C, 53 differential metabolites (*p* < 0.05, VIP > 1) were detected, including 23 metabolites enriched in the HTN-AF group and 30 in the HTN group. In addition, Spearman’s correlation analysis between the significantly distinct metabolites and discriminant genus identified by LEfSe analysis suggested that altered metabolites enriched in the HTN-AF group were positively correlated with genera enriched in the HTN-AF group, but negatively correlated with genera enriched in the HTN group (Figure 5D), revealing the potential influence of GM dysbiosis on host metabolome. 

### 3.6. Unbalanced Saturated and Unsaturated Fatty Acids in HTN Patients with AF

**Figure 6:**
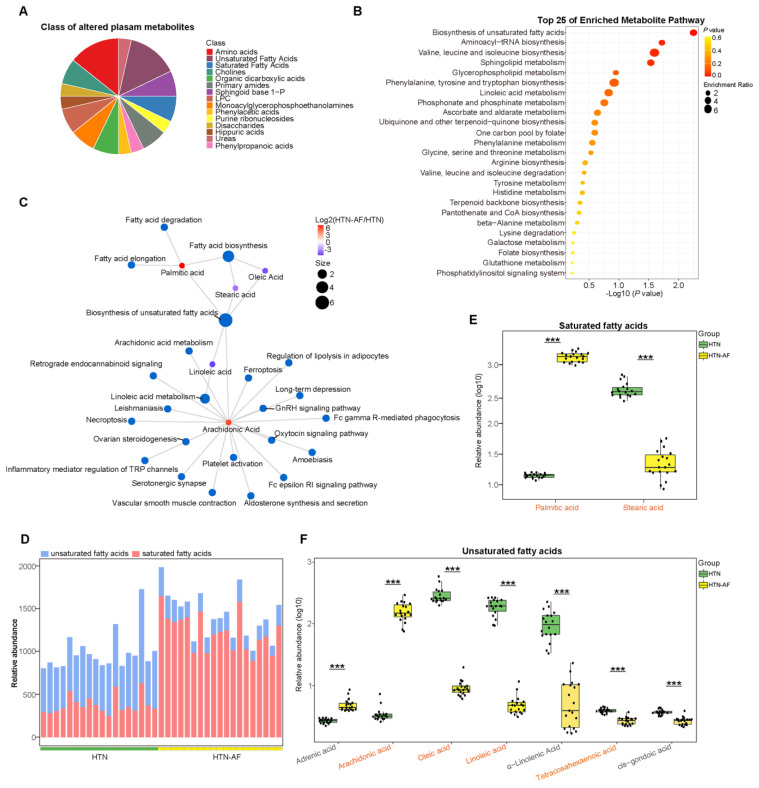
Aberrant distribution of saturated and unsaturated fatty acids in HTN-AF patients. (**A**) Pie chart depicting the class proportion of differential metabolites in the circulation of the subjects. (**B**) Bubble chart of top 25 pathway (class: metabolism) enrichment of distinct metabolites between the two groups. (**C**) Network chart illustrating the KEGG pathway (all classes) involved in differential fatty acids. (**D**) Stack graph showing the composition ratios of saturated and unsaturated fatty acids for participants. Blue, unsaturated fatty acids; red, saturated fatty acids. (**E**) Box plots showing the relative abundance of two saturated fatty acids, including palmitic acid and stearic acid, in the HTN (green) and HTN-AF (yellow) groups. *t*-test. (**F**) Box plots showing the relative abundance of seven unsaturated fatty acids, including adrenic acid, arachidonic acid, oleic acid, linoleic acid, α-linolenic acid, tetracosahexaenoic acid, and cis-gondoic acid, in the HTN (green) and HTN-AF (yellow) groups. *t*-test; orange-colored word, VIP > 1; ***, *p* < 0.001.

Based on the KEGG database, altered serum metabolites were annotated to the enriched metabolic pathway, and Figure 6B listed the top 25, especially biosynthesis of unsaturated fatty acids. Among the 15 classes divided by the differential metabolites, saturated and unsaturated fatty acids accounted for the most significant proportion, at 14.29% (Figure 6A), suggesting an increasing disturbance in fatty acid homeostasis in HTN-AF patients. This study detected the circulation of a total of two saturated fatty acids, including palmitic acid (PA) and stearic acid (SA), and seven unsaturated fatty acids, including arachidonic acid (AA), tetracosahexaenoic acid, oleic acid (OA), linoleic acid (LA), adrenic acid, α-linoleic acid (ALA), and cis-gondoic acid. As shown in Figure 6D, the composition ratios of saturated fatty acid and unsaturated fatty acid were different between the two groups. Based on *t*-test (*p* < 0.05) and OPLS-DA (VIP > 1), two saturated fatty acids and four unsaturated fatty acids were identified as differential metabolites (Figure 6E,F). Compared with HTN patients, PA and AA were significantly elevated in HTN-AF patients, while tetracosahexaenoic acid, OA, LA, and SA were dramatically decreased (all *p* < 0.001, VIP > 1) (Figure 6E,F). In addition, increased levels of adrenic acid and reduced levels of ALA and cis-gondoic acid were also observed in HTN-AF patients (*p* < 0.001, VIP < 1). Subsequently, the significance of saturated/unsaturated fatty acids was further tested through the linear model with covariate adjustment for age, TC, DM, and male gender, confirming that except for adrenic acid (*p* = 0.134), the *p* values for other saturated/unsaturated fatty acids—including PA, SA, AA, OA, LA, tetracosahexaenoic acid, ALA, and cis-gondoic acid—were all less than 0.001 (Table S2). The co-occurrence network was constructed for the KEGG pathways (all classes) and these distinctly differential saturated/unsaturated fatty acids (Figure 6C) to further evaluate their functions in host homeostasis. Notably, AA, enriched in the HTN-AF group, interacted with other differential fatty acids and contributed to multiple inflammatory and pathogenic pathways, including Fc epsilon RI signaling pathway, Fc gamma R-mediated phagocytosis, aldosterone synthesis and secretion, platelet activation, ferroptosis, and necroptosis, etc. [19,20,21,22,23,24,25,26]. 

### 3.7. A Risk Prediction Model based on Gut Microbial Signatures for AF in HTN

**Figure 7:**
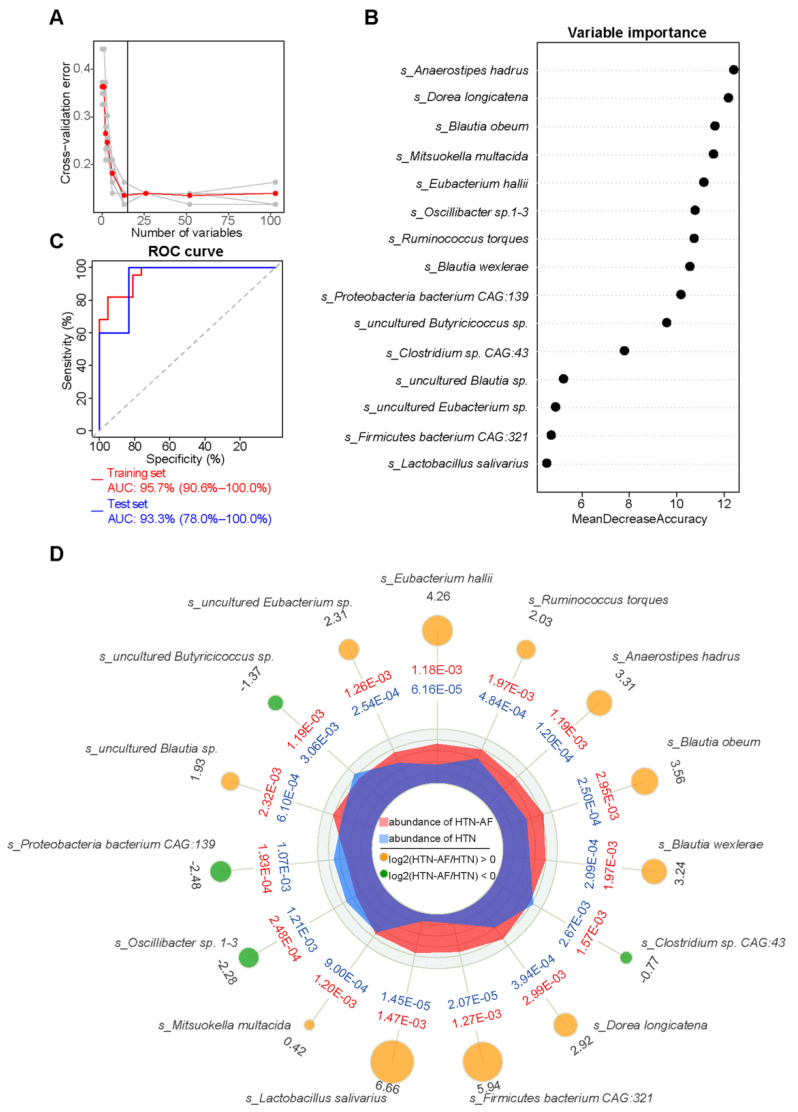
A species-based random forest model for predicting AF occurrence in HTN patients. (**A**) The random forest disease classifier, trained on the relative abundance of the species in the HTN and HTN-AF as variables, describing the distribution of five trials of 10-fold cross-validation error and the number of species in the optimal set with the lowest cross-validation error. (**B**) The variable importance of selected species. (**C**) Receiver-operating characteristic curve (ROC) analysis on the training (AUC = 95.7%, 95% CI: 90.6–100.00%) and test sets (AUC = 93.3%, 95% CI: 78.0–100.00%). (**D**) Radar chart showing the mean and log2 fold-change in the relative abundance between the two groups (All *p* < 0.05, Wilcoxon rank sum test).

**Figure 8:**
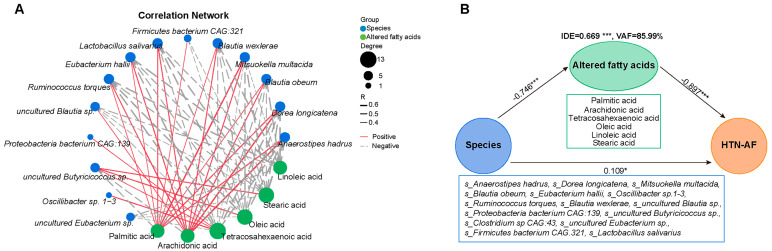
Association between GM dysbiosis and altered saturated and unsaturated fatty acids in HTN patients with and without AF. (**A**) Correlation network plot depicting the significant correlations between selected species based on random forest analysis and six different saturated/unsaturated fatty acids in the two groups (all *p* < 0.05). Spearman’s correlation analysis. (**B**) The partial least squares structural equation modeling (PLS-SEM) model shows the mediation effect of six altered fatty acids in the total effect of gut species on AF occurrence in HTN patients. Path coefficients were indicated, and the variance accounted for (VAF) score was 85.99%. *, *p* < 0.05, ***, *p* < 0.001.

Subsequently, a prediction model was established to assess the risk of AF occurrence in HTN patients individually. Using the random forest classifier (Figure 7A), 15 characteristic species were detected to distinguish AF occurrence in HTN with a high predictive value (AUC = 93.3%, 95% CI: 78.0–100.0%) (Figure 7C), including *Anaerostipes hadrus*, *Dorea longicatena*, *Blautia obeum*, *Mitsuokella multacida*, *Eubacterium hallii*, *Oscillibacter* sp.1–3, *Ruminococcus torques*, *Blautia wexlerae*, *Proteobacteria bacterium CAG:139*, *uncultured Butyricicoccus *sp., *Clostridium sp. CAG:43*, *uncultured Blautia *sp., *uncultured Eubacterium *sp., *Firmicutes bacterium CAG:321*, and *Lactobacillus salivarius.* The specific importance and relative abundance of these selective species were presented in Figure 7B,D. Spearman’s correlation analysis and correlation network were used to illuminate the significant associations between the represented species and differential fatty acids (Figure 8A). Some HTN-AF-enriched species (e.g., *Anaerostipes hadrus*, *Dorea longicatena*, *Blautia obeum*, *Mitsuokella multacida*, *Eubacterium hallii*, *Ruminococcus torques*, *Blautia wexlerae*, *uncultured Blautia *sp., *Firmicutes bacterium CAG:321*, and *Lactobacillus salivarius*) were positively correlated to AA and PA, while negatively related to fatty acids decreased in the HTN-AF group (such as tetracosahexaenoic acid, OA, LA, and SA) (Figure 8A) (*p* < 0.05). Moreover, the mediation analysis revealed the relationship among species, altered fatty acids, and AF in HTN patients (Figure 8B). The total, direct, and indirect association of these species with AF were confirmed in HTN patients, and altered fatty acids significantly mediated the indirect effects of gut species on AF, with an 85.99% proportion of mediating effects. These findings revealed the underlying predictive potential and influence of GM on AF occurrence in HTN patients.

## 4. Discussion

This study comprehensively assessed gut microbial composition and function and relevant circulating metabolites in HTN patients with and without AF, revealing significant alterations in GM and metabolic patterns in HTN patients comorbid with AF. The coexistence of HTN and AF increased alpha-diversity, accompanied by an enterotype shift from *Prevotella* to *Bacteroides*, suggesting the overgrowth and aggravated disturbance of GM. Further analysis of gut microbial metabolic patterns and circulating metabolites by the KEGG database and serum metabolomics demonstrated the dramatic difference between HTN patients with and without AF, which were closely associated with GM dysbiosis. In particular, saturated and unsaturated fatty acids, including AA, PA, tetracosahexaenoic acid, OA, LA, and SA, significantly changed in HTN-AF patients and mediated 85.99% indirect effects of GM on AF occurrence. Moreover, a random forest model based on species signature profiles was established to predict the AF risk in HTN patients. Disordered GM, coupled with an imbalance of circulating GM-related metabolites, was linked to AF occurrence in HTN and may be a promising target for intervention.

As a complex and enormous ecosystem, GM produces a considerable amount of microbial metabolites that are absorbed into the circulation and act on target organs to influence host homeostasis and the progression of diseases, including cardiovascular disease (CVD) [27]. Accumulating studies have demonstrated that disordered GM and relevant metabolites imbalance are the co-promoter of AF and HTN and thus may mediate the AF occurrence in HTN patients [6,7]. Strikingly, this study indicated further increased gut microbial diversity and altered composition in patients with HTN and AF coexisting. Some bacteria were significantly accumulated in the gut of HTN-AF patients, including genera *Ruminococcus*, *Streptococcus*, *Veillonella*, *Dorea*, and *Enterococcus.* Previous studies have reported that *Ruminococcus, Streptococcus,* and *Dorea* possess pro-inflammatory properties, promoting the production of inflammatory factors [13,14,15,16,17,18]. For example, *Ruminococcus* enhances the levels of interferon-γ (IFNγ), interleukin (IL)-17, and IL-22 in germ-free and is abundant in the gut in patients with Alzheimer’s disease (AD) and obstructive sleep apnea-hypopnea syndrome (OSAHS) [14,15,16]; *Streptococcus* is associated with the production of IFNγ and IL-1β and is elevated in patients with HTN, AF, congestive heart failure (CHF), and atherosclerotic cardiovascular disease (ACVD) [17,18]; and a subset of *Dorea* species has also been associated with the release of IFNγ [18], especially *Dorea longicatena*, which is abundant in HTN-AF patients in the present study. Inflammation, as a critical role in the pathogenesis of AF, can alter atrial electrical and structural substrates and calcium homeostasis, thereby resulting in increased vulnerability to AF [28]. Subsequently, a species-based random forest prediction model was constructed to help identify HTN patients with high susceptibility to AF, with a certain predictive value. 

Furthermore, altered GM functional patterns and circulating metabolites were also observed in HTN-AF patients. As a virtual endocrine organ, GM-host interactions are mainly mediated through many metabolism-dependent pathways and the generation of bioactive metabolites [29]. Some GM-related metabolism pathways were abundant in the gut of HTN-AF patients, including linoleic acid metabolism, arachidonic acid metabolism, and secondary bile acid biosynthesis, etc. Notably, serum metabolomics revealed that the aggravated imbalance of saturated and unsaturated fatty acids was associated with AF occurrence in HTN patients, including significant enrichment of PA and AA and decreased levels of OA, LA, tetracosahexaenoic acid, and SA. OA and LA are known to inhibit the production of reactive oxygen species (ROS) [30,31], while AA is known to stimulate ROS production [32]. Meanwhile, PA, the most abundant type of saturated fatty acid in the human body, induces inflammation, oxidative stress, and apoptosis in cardiomyocytes [33], which are all thought to be involved in forming AF-perpetuating substrates [34]. In a community-based longitudinal cohort, a higher risk of AF is positively correlated to circulating PA and negatively related to SA [35]. This finding also applied to HTN patients in our study. The interaction of differential fatty acids was involved in multiple KEGG pathways, including Fc epsilon RI signaling pathway, Fc gamma R-mediated phagocytosis, aldosterone synthesis and secretion, ferroptosis, and necroptosis, etc., which were recognized as inflammation-related signaling pathways and were related to the pathogenesis of AF [14,15,16,17,18]. This evidence tentatively indicated that the aggravated dysregulation of GM and related metabolites in HTN was involved in the development of AF and may be an underlying preventative target.

However, there are some limitations in the present study. This study was cross-sectional with a small sample size, which may lead to selection bias, and lacked relevant mechanism exploration. Follow-up prospective cohorts with expanded sample sizes and mechanistic research are required in the future to translate gut microbial therapy into clinical applications. 

## 5. Conclusions

The current study revealed that AF occurrence in HTN patients was correlated to exacerbated dysbiosis in GM characteristics and circulating metabolite profiles, providing fundamental evidence for further exploration on the interaction between disordered GM and AF susceptibility in HTN. 

## Figures and Tables

**Table 1:** Baseline clinical characteristics of the HTN patients with or without AF.

	HTN	HTN-AF	*p* Value
Number	27	27	
Male (%)	26 (96.30)	18 (66.67)	0.011
DM (%)	0 (0.00)	7 (25.93)	0.010
BMI, kg/m^2^	25.80 ± 3.06	27.10 ± 5.56	0.298
Age, years	53.59 ± 4.89	68.74 ± 8.40	<0.001
TC, mmol/L	4.59 ± 1.04	3.89 ± 1.11	0.020
TG, mmol/L	1.43 ± 0.87	1.67 ± 1.53	0.478
LDL, mmol/L	3.06 ± 3.27	2.16 ± 0.97	0.176
ALT, U/L	21.56 ± 9.65	21.67 ± 11.10	0.971
sCr, μmol/L	70.00 (59.75, 92.54)	69.30 (59.00, 86.30)	0.663
FBG, mmol/L	5.29 (4.55, 5.71)	4.79 (4.50, 6.01)	0.961

Data are presented as mean ± SD, median (quartile), or number (%). ALT, alanine aminotransferase; BMI, body mass index; DM, diabetes mellitus; FBG, fasting blood glucose; LDL, low-density lipoprotein; sCr, serum creatinine; TC, total cholesterol; TG, triglyceride.

## Data Availability

The data supporting this work have been deposited in the EMBL European Nucleotide Archive (ENA) under the BioProject accession code PRJEB28384. The serum metabolomics data are available at the NIH Common Fund’s Data Repository and Coordinating Center website with Metabolomics Workbench study ID: ST001169.

## Supplementary Materials

The following supporting information can be downloaded at: https://www.mdpi.com/article/10.3390/biom12101445/s1, Figure S1: multivariate analysis by linear models (MaAsLin) adjusted for potential confounders (total cholesterol, gender, diabetes mellitus, and age) showed remarkable differences in GM between the two groups; Figure S2: ANOSIM analysis of KEGG orthology between the HTN and HTN-AF groups; Table S1: results of multivariate analysis by linear models; Table S2: results of the linear model with covariate adjustment.
