# Supplementary material for: Aggravated Gut Microbiota and Metabolomic Imbalances Are Associated with Hypertension Patients Comorbid with Atrial Fibrillation
Source: Biomolecules. 2022 Oct 9;12(10):1445. doi: 10.3390/biom12101445 (PMC9599445; doi:10.3390/biom12101445)
Supplement: Supplementary file 1 [file biomolecules-12-01445-s001.zip › biomolecules-1922397-supplementary.pdf]

Table S1

Results of Multivariate Analysis by Linear Models.

Variables	Coefficient	Correlation direction	*P* value
g_Eubacterium	0.068	Positive	<0.001
g_Blautia	0.068	Positive	<0.001
g_Ruminococcus	0.041	Positive	0.018
g_Dorea	0.039	Positive	<0.001
g_Bifidobacterium	0.034	Positive	0.045
g_Fusicatenibacter	0.030	Positive	<0.001
g_Anaerostipes	0.023	Positive	<0.001
g_Streptococcus	0.021	Positive	<0.001
g_Lachnospira	0.017	Positive	<0.001
g_Coprococcus	0.014	Positive	0.008
g_Coprobacillus	0.014	Positive	<0.001
g_Veillonella	0.013	Positive	0.002
g_Romboutsia	0.013	Positive	0.001
g_Dialister	0.011	Positive	0.200
g_Tyzzerella	0.009	Positive	<0.001
g_Intestinibacter	0.009	Positive	<0.001
g_Enterococcus	0.007	Positive	<0.001
g_Lactobacillus	0.007	Positive	<0.001
g_Eggerthella	0.006	Positive	0.022
g_Clostridioides	0.006	Positive	0.006
g_Klebsiella	0.005	Positive	0.433
g_Butyrivibrio	0.005	Positive	0.020
g_Holdemanella	0.005	Positive	<0.001
g_Shigella	0.004	Positive	0.036
g_Citrobacter	0.003	Positive	0.020
g_Mycoplasma	0.002	Positive	0.006
g_Turicibacter	0.002	Positive	0.008
g_Mitsuokella	0.002	Positive	<0.001
g_Azospirillum	−0.001	Negative	0.063
g_Pseudoflavonifractor	−0.005	Negative	0.001
g_Paraprevotella	−0.005	Negative	0.028
g_Ornithobacterium	−0.006	Negative	<0.001
g_Intestinimonas	−0.007	Negative	0.023
g_Parasutterella	−0.012	Negative	0.005
g_Flavonifractor	−0.014	Negative	0.006
g_Sutterella	−0.014	Negative	0.181
g_Bilophila	−0.015	Negative	0.067
g_Butyricicoccus	−0.017	Negative	<0.001
g_Oscillibacter	−0.052	Negative	0.005
g_Prevotella	−0.142	Negative	0.025

Confounders: age, diabetes mellitus (DM), total cholesterol (TC) and male gender.

Table S2

Results of the linear model with covariate adjustment.

Variables	β	*P* value
Palmitic acid	17.05	<0.001
Tetracosahexaenoic acid	10.78	<0.001
Linoleic acid	8.75	<0.001
Arachidonic acid	7.31	<0.001
Oleic acid	3.25	<0.001
Stearic acid	3.05	<0.001
α-Linolenic acid	1.21	<0.001
cis-gondoic acid	0.59	<0.001
Adrenic acid	−4.66	0.134

Covariate: age, diabetes mellitus (DM), total cholesterol (TC) and male gender.

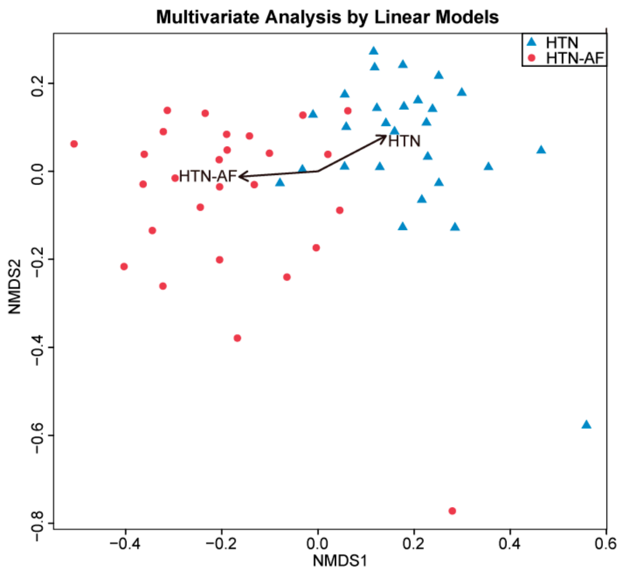

Figure S1. Multivariate Analysis by Linear Models (MaAsLin) adjusted for potential confounders (total cholesterol, gender, diabetes mellitus, and age) showed remarkable differences in GM between the two groups.

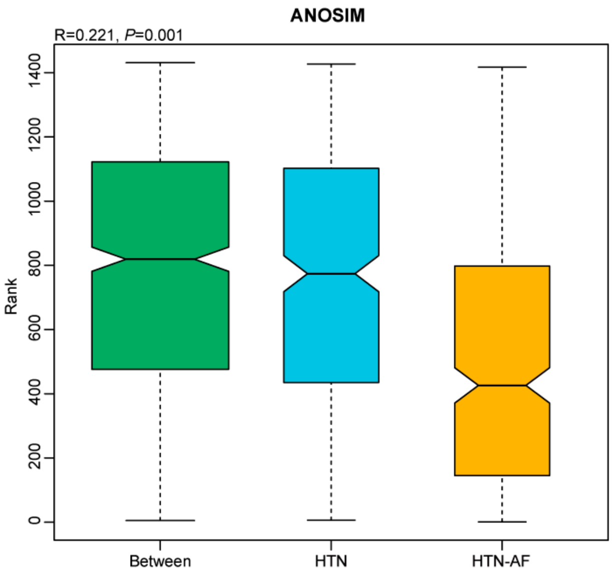

Figure S2. Anosim analysis of KEGG orthology between the HTN and HTN-AF groups.
